# Functional assessment of current upper limb prostheses: An integrated clinical and technological perspective

**DOI:** 10.1371/journal.pone.0289978

**Published:** 2023-08-16

**Authors:** Patricia Capsi-Morales, Cristina Piazza, Lis Sjoberg, Manuel G. Catalano, Giorgio Grioli, Antonio Bicchi, Liselotte M. Hermansson

**Affiliations:** 1 School of Computation, Information and Technology, Technische Universität München, Garching, Germany; 2 School of Health Sciences, Örebro University, Örebro, Swede; 3 Instituto Italiano di Tecnologia, Genoa, Italy; 4 Centro “E. Piaggio” and Dipartimento di Ingegneria dell’Informazione, University of Pisa, Pisa, Italy; 5 University Health Care Research Centre, Faculty of Medicine and Health, Örebro University, Örebro, Sweden; Polytechnic University of Marche: Universita Politecnica delle Marche, ITALY

## Abstract

Although recent technological developments in the field of bionic upper limb prostheses, their rejection rate remains excessively high. The reasons are diverse (e.g. lack of functionality, control complexity, and comfortability) and most of these are reported only through self-rated questionnaires. Indeed, there is no quantitative evaluation of the extent to which a novel prosthetic solution can effectively address users’ needs compared to other technologies. This manuscript discusses the challenges and limitations of current upper limb prosthetic devices and evaluates their functionality through a standard functional assessment, the Assessment of Capacity for Myoelectric Control (ACMC). To include a good representation of technologies, the authors collect information from participants in the Cybathlon Powered Arm Prostheses Race 2016 and 2020. The article analyzes 7 hour and 41 min of video footage to evaluate the performance of different prosthetic devices in various tasks inspired by activities of daily living (ADL). The results show that commercially-available rigid hands perform well in dexterous grasping, while body-powered solutions are more reliable and convenient for competitive environments. The article also highlights the importance of wrist design and control modality for successful execution of ADL. Moreover, we discuss the limitations of the evaluation methodology and suggest improvements for future assessments. With regard to future development, this work highlights the need for research in intuitive control of multiple degrees of freedom, adaptive solutions, and the integration of sensory feedback.

## 1. Introduction

The adult rejection rate for myoelectric and body-powered upper-limb prostheses are estimated at 23% and 27%, respectively [1]. It is considered that 20% of persons with limb deficiency do not try any active or passive solution. The limited users’ acceptance represents a huge problem in commercially-available prostheses and emphasizes that something is still missing in the provided solutions. Moreover, there is a strong discrepancy between research solutions for artificial prosthetic limbs and the technology used by prosthesis users in real life [2]. The challenges are multiple and intricate. The direction followed by many research groups often focuses on the development of advanced and sophisticated systems, that do not always meet users’ needs for intuitiveness and reliability. A promising approach to obtaining seamless natural–artificial integration consists of combining advanced mechatronic solutions for dexterous and highly sensorized robotic hands with novel methods for robust and effective interfaces [3]. Not only the solutions must be dexterous, but they may be robust, reliable and accessible products for users, and they require complete assessments from therapists to evaluate and explore new available features. A major multidisciplinary work of engineers, researchers, clinicians, and prosthesis users is necessary to define future trends and requirements of the new generation of prostheses.

Generally, there is no consensus on surveys regarding desired prosthesis by users. A survey of European and American amputees [4] noted that the most desired functional features are the ability to move separate fingers, avoiding the slipping of grasped objects and proportional grip strength, while [5] suggested interest in soft and adaptable solutions. Users of prosthetic upper limbs also conveyed the need for an increase range of motion and speed of the wrist movements, a more natural appearance and sensory feedback. Other critical aspects are related to the socket design (comfort, temperature and transpiration management) and reductions in terms of weight and noise [6, 7]. However, these aspects may not reflect the priorities of non-expert myoelectric users, where preferences are more humble [8].

Analysis of the manipulation strategies employed by upper-limb prosthesis users devices can yield valuable scientific insights into the limitations of existing prosthetic technology. Traditionally, this problem has been addressed through surveys or laboratory-based studies, which tend to focus on prehensile grasp-related outcomes and may not accurately reflect the complexities of daily activities, as previously indicated by [9]. This work proposes a “quantitative” analysis and review of new trends and current challenges in upper limb prostheses. As a representation of the state-of-the-art, we selected the participants of Cybathlon Powered Arm Prostheses Race [10], an international competition that addresses unresolved challenges encountered in everyday life, with the primary objective of establishing a benchmark for the latest advancements in research laboratories and companies worldwide. There, upper-limb prosthetic technologies must support users to complete 6 tasks based on Activities of Daily Living (ADL). This platform offers the unique opportunity to assess different technical solutions under the same conditions. Although the main metric used for the Cybathlon competition is based on the time to complete the race, we aim to analyze the performance of each team using a standard clinical assessment. Earlier studies on Cybathlon offer valuable perspectives on the influential role of this event in advancing the field of assistive technology and research impact [10, 11]. Additionally, regarding the Powered Arm Prostheses Race, the preparatory activities associated with the Cybathlon competition have promoted a human-centered design approach [12, 13] and have facilitated an effective test bench for assessing system performance in real-world settings [14].

In this work, we examine the behavior of prosthesis users in unstructured and familiar environments, specifically focusing on data obtained from Cybathlon participants and their corresponding video footage. Multiple video cameras were utilized to capture external views of the participants’ arms and hands as they engaged in specific activities. A total of 7 hours and 41 minutes of video footage were recorded during the competition, involving 23 individuals with unilateral amputation or limb difference who employed 23 distinct technologies. All video data underwent analysis by four certified expert observers using a functional assessment that consists of 22 categories. This analysis generated tags indicating the type of manipulation performed, the duration of both prehensile and non-prehensile manipulations, and other observations. This approach enabled an objective analysis of multiple systems under consistent usage conditions and assessment procedures, an unprecedented advancement in studying this population.

The Assessment of Capacity for Myoelectric Control (ACMC) [15], which is the first functional assessment created specifically for upper limb prostheses, is used to score prosthesis user behavior during the Cybathlon tasks, due to their similarity with ADL. The ACMC framework permits the assessment of technology functionality in a wide range of environments that emulate everyday activities, independent of the specific materials or objects used. Here, the evaluation generated by the ACMC framework empowers this comparison with a supplementary assessment with a clinical perspective. Note that this work do not relay on existing data but computes the ACMC score for each technological solution. For completeness, we also include a detailed analysis of competition results and a deep development prospect for upper limb prostheses. However, it is not the scope of this paper to analyze or discuss Cybathlon as a competition or event, but employ it as a controlled environment for further clinical analysis and comparison of technologies. To the best of the authors knowledge, this is the first evaluation of the state-of-art that offers not only an indication of the technical specifications but provides a funtional assessment performed by certified therapists.

## 2. Current situation

In recent years, remarkable progress has been made in the development of upper limb prostheses, driven by the growing need for enhanced functionality and usability. This surge in innovation has been propelled by research laboratories and companies worldwide, seeking to address the challenges faced by individuals with limb loss. Before delving into the detailed analysis of the different technologies included in this study, it is important to provide an overview of the current landscape of upper limb prosthetic solutions. This section presents a comprehensive examination of various trends, ranging from commercially available options to clinical solutions, and even prototype technologies that are still in the realm of research exploration. By offering a holistic perspective of the state-of-the-art, this work aims to identify the main current developments in the field and shed light on the advancements made across the spectrum of prosthetic solutions.

Commercially available solutions can be divided into three categories: passive cosmetic limbs, mechanical grippers and more advanced robotic prosthetic hands. Cosmetic solutions are often used for partial hand amputations (e.g., fingers) while active prostheses are adopted by patients with a more proximal level of limb loss. Among active prostheses, body-powered mechanical hooks are usually preferred for their low price, light weight, and easy maintenance. The actuation mechanism of these devices is based on a cable that encompasses one or both shoulders to control one DOF, and also provide inherent proprioception feedback. This type of prosthesis is also well suited for high-intensity work due to its control robustness. Robotic prosthetic hands potentially could offer the most versatile, natural, and power-efficient replacement. In contrast to body-powered, myoelectric prostheses are externally powered and must be recharged regularly. In these devices, control approaches based on surface electromyography (sEMG) sensors are by far the most widely used technique to decode motor intentions. The vast majority of commercially available self-powered prostheses use simple threshold-based sEMG decoding, with few surface sensors to control one DOF [16]. In some cases, the system allows more DOFs, but this comes at the cost of a non-intuitive command scheme [17]. Furthermore, the price of advanced robotic solutions is an additional limiting factor for broader adoption by patients.

Among emerging trends in research and promising design options [18], under-actuation is a widespread approach to simplify mechanics while keeping reasonable dexterity. An under-actuated system is one where the number of degrees of actuation (DoAs) is smaller than the number of degrees of freedom (DoFs). While rigid architectures are still the norm in commercial prostheses, there has been recent interest in the development of flexible systems [8, 19]. Decades of research on myoelectric prostheses has led to numerous invasive and non-invasive solutions for interfacing with body signals. Advances in control strategies can be described in terms of decoding type (e.g., classification or continuous control) and functional achievement (e.g., number of DOFs, grasping, or single-finger decoding). A growing interest in machine learning techniques has resulted in promising methods to interpret user’s intentions from non-invasive interfaces [20]. Implanted EMG solutions, which use sensors surgically inserted into residual muscles, have shown higher performance and stability than sEMG on the continuous control of up to three DOFs [21]. Electrode invasiveness, signal quality and stability are important aspects that have been extensively studied, both for the physical interface, and the filtering process [22].

An additional challenge consists of the biomechanical integration of artificial limbs with the body. Although recent advancement in terms of materials and the high customization level of these technologies, current options for the socket remains highly unsatisfactory for patients [23, 24]. A promising clinical alternative is the attachment via a direct connection to the residual skeletal structures, termed osseointegration [25, 26]. This represents a stable physical connection and avoids pressure on the soft tissues of the residual limb, with a consequent reduction in discomfort and pain.

An additional important component consists of a feedback systems, that can increase both acceptability and performance of the new generation of robotic prosthetic hands [27–29]. Due to the lack of sensory feedback in most commercial robotic hands, patients usually rely on constant visual inputs, which might be tiring, prone to error and unnatural. Tactile feedback using vibrotactile [30, 31], mechanotactile [32], or sensory substitution has been proposed by several researchers, through invasive or non-invasive solutions. Surgically implanted electrodes have shown levels of sensory recovery is far superior to those of non-invasive approaches [3] in terms of functionality, intuitiveness and required cognitive load. The intimate prosthesis-skeletal junction achieved with osseointegration also allows users to experience improved pressure and vibratory sensation [33] for sensory feedback recovery [34, 35].

## 3. Methods

Given the increasing complexity of novel robotic prosthetic hands that may integrate both sensory and motor functionalities, an appropriate rehabilitation process is considered fundamental. For this reason, it is important to have standardized tools to measure the effectiveness of novel technologies and compare different approaches on a common metric. This is a very challenging task as it requires access to multiple technological solutions, patients and common assessment methods. In this work, we used the participants of the Cybathlon competition as a representation of the state of the art of prostheses, both for commercial and research solutions. We conducted a comprehensive evaluation of various technologies using the ACMC, the first standard assessment specifically designed for upper limb prostheses. The ACMC allows for the evaluation of a wide range of tasks inspired by ADL, and provides detailed analysis of all stages of manipulation, including grasping, holding, and releasing. In addition, we present an overview of the Cybathlon race results related to the technology used.

### 3.1 Assessments of prosthetic solutions

To date, the most frequently used standard functional assessments were developed for neurological impairments (such as a stroke), and have been adapted for the evaluation of prosthetic limbs. Generally, they evaluate the capability of a person to perform specific movements or succeed in a single repeated grasping action, for example: Box and Block Test of manual dexterity [36], Southampton Hand Assessment Procedure (SHAP) [37] and Jebsen-Taylor Hand Function Test [38]. However, assessing the performance of upper limb prostheses involves considering multiple aspects that collectively contribute to the overall functionality and usability of the prosthetic system. Evaluating these aspects individually can be not only challenging but may not provide a comprehensive understanding of the system’s performance in real-world conditions. For instance, the authors in [9] conducted a study wherein head-mounted video cameras were employed to capture egocentric perspectives of the hand and terminal device of individuals with unilateral upper-limb-difference while they performed various household tasks for several hours in their own homes, in the absence of an experimenter.

Only a few outcome measures focus on a complete and controlled evaluation of performance in daily tasks, which can provide information on several aspects, including acceptance and integration of the prosthesis into everyday life needs. Among them, the Assessment of Capacity for Myoelectric Control (ACMC) is an observational-based assessment that measures a person’s ability to operate a myoelectric prostheses when performing activities of daily living [15]. The ACMC evaluates hand functionalities through the assessment of different aspects in manipulation. To ensure a precise and systematic evaluation, this study focuses on the aspects evaluated in the ACMC. Main factors of evaluation are: the need for external support, control of grip force, coordination of both hands, ability to grasp proficiently in different positions and in motion (which considers the execution time), repetitive grasp and release capabilities, and the need for visual feedback. The test consists of 22 items divided into 4 subsections (Gripping, Re-adjusting Grip, Holding, Releasing) scored on a 4-point rating scale by a certified observer. Items differ in movement and level of difficulty. Each item has a definition that guides the rater to identify the moment in which it occurs and its quality (from *not capable* to *extremely capable*). Furthermore, the minimal detectable change (MDC) indicates whether a change between ACMC scores is due to measurement error or an actual change in performance between technologies, and it is used in this work. The MDC is 2.5 ACMC units for the same rater and 3.1 ACMC units for different raters. Since the engagement of the subject is a fundamental requirement to achieve reliable results, the testing environments are inspired by activities of daily living and hobbies and should be selected according with user’s preferences. It is recommended to keep the subject motivated during the evaluation, which is assumed by the competition, and to accomplish the tasks spontaneously in their usual way. The ACMC proved to be a valid, reliable and sensitive evaluation of qualitative aspects of myoelectric control and hand features [15].

### 3.2 CYBATHLON competition

Cybathlon is an international competition normally held every four years, that promotes the development of assistive technologies with and for people with disabilities. Each team, consisting of technology developers from universities, companies or NGOs and a person with disability (referred to as pilot), tackles various everyday tasks with a proposed assistive technology. Among the 6 disciplines, the Powered Arm Prosthesis Race (ARM race) focuses on grasping, holding and manipulation tasks with different levels of difficulty. In the first two Cybathlon editions (2016 and 2020), the ARM race consists of completing 6 tasks (for a full description, please refer to S1 Appendix) within a limited time of 8 minutes. To promote the use of the assistive device, objects colored in blue can only be grasped with the prostheses. Note that tasks included in Cybathlon 2016 were slightly different, even in their order, from those included in 2020.

Cybathlon 2016 was an in-person event and the official results (S1 Fig) are the score and time obtained in the finals A, B and C. The 10 participants were divided into these 3 groups according to the results of a qualification round. Because of the pandemic, Cybathlon 2020 Global Edition was an online event and all 13 participant teams had to compete at their venue. They had three opportunities to complete the race and only the best one (highest score and fastest time) was considered for the final classification. S2 Fig shows the ranking of all teams for the three races included in Cybatlon 2020 Global Edition.

**Fig 1:**
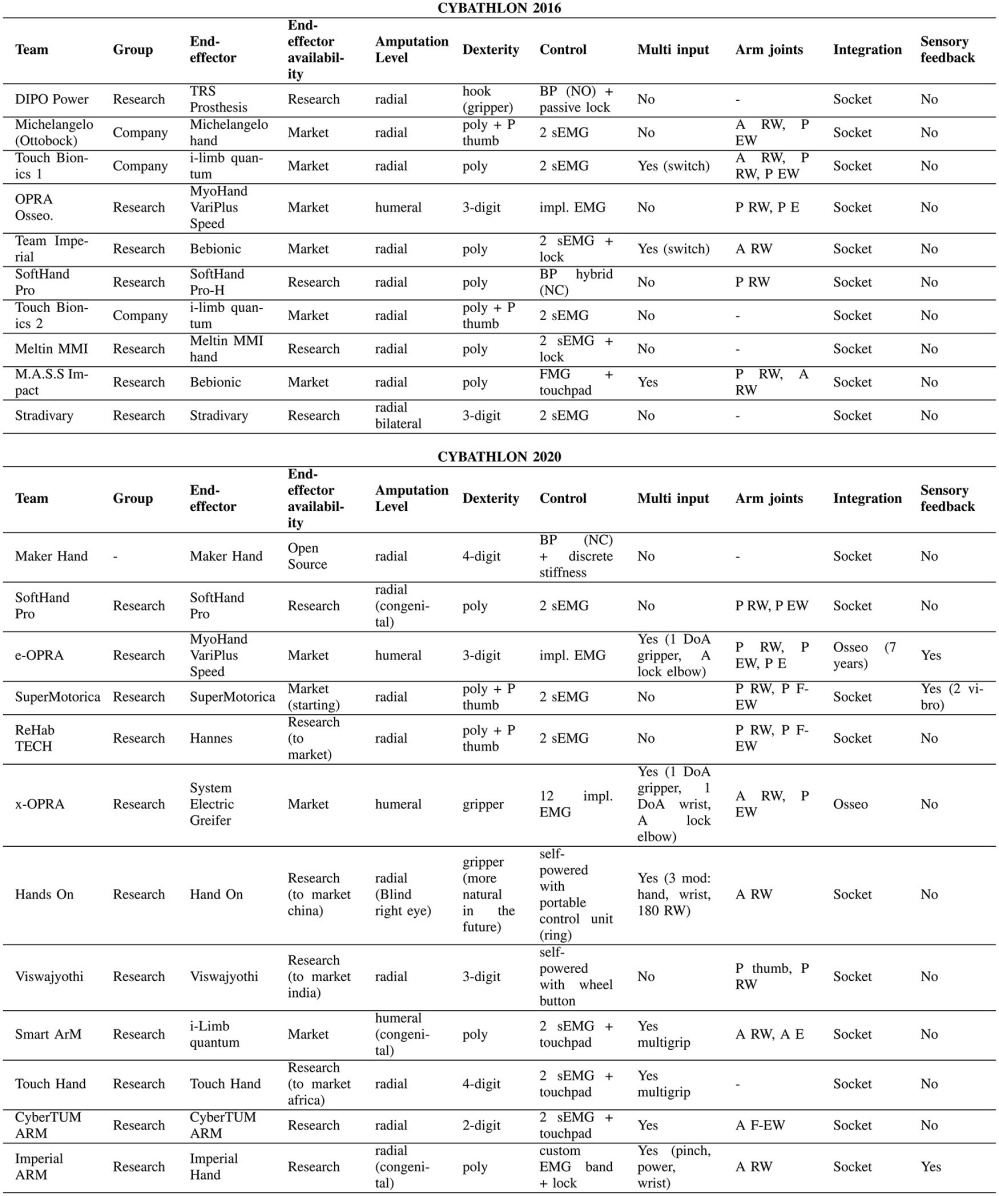
Specifics from participants of Cybathlon 2016 and 2020. (A) refer to active and (P) to passive joints. Note that (RW) rotational wrist, (EW) extension wrist, (F-EW) flexion-extension wrist and (E) elbow. For control modalities, (BP) refers to Body-powered with (NC) normally closed and (NO) normally opened; (EMG) refers to electromyography sensors, and (FMG) to force myography sensors and (imp. EMG) to implanted EMG sensors.

To participate in the ARM race, pilots must have an amputation or congenital malformation above the wrist or higher, on at least one arm. Body-powered or self-powered prostheses that are operated completely in manual mode or include autonomous functions are allowed. The prosthetic device can include any number of actively driven, passive or mechanically coupled joints, e.g. at the fingers. Fig 1 presents data and characteristics from the participants in both events. It is visible how the ARM race includes commercial devices and research prototypes ranging from grippers to more anthropomorphic advanced hands, providing a collection of systems that cover most of the state-of-art solutions.

## 4. Results

### 4.1 Specifics of prostheses representation

There is an overall participation of subjects with different levels of amputation (see Fig 1), including 17.4% of transhumeral, 82.6% transradial and 1 bilateral subject. Note that at the transradial level, different lengths of the stump may strongly affect the movement of the user, and may restrict or not wrist movements. Furthermore, 23% of the participants in 2020 were subject with congenital limb deficiency.

S1 and S2 Figs show images of the technologies that have participated in Cybathlon 2016 and Cybathlon 2020, respectively, and that are included in this analysis. Since 2016, the participation has also evolved from commercially-available hands (from 60% to 34.6%) to research system (from 40% to 65.4%), including three groups from developing countries (India, China and Africa), which propose research systems that may enter in their corresponding markets. Regarding body-powered solutions, there is a shift from hook-like solutions to more advanced mechanical hands, including an additional feature to select the level of force applied. Both editions presented subjects with osseointegration and implanted EMG sensors, but only *e-OPRA* from 2020 also included sensory feedback. Some hands embed a compliant flexion-extension wrist, but only one team proposed a complete soft-robotic hand with elasticity in all the fingers. Moreover, under-actuation is also present among the participants; some systems present more complex actuation architecture and others only in the finger joints. However, in 2020 this concept gets higher interest, representing 30% (instead of only 10% in 2016). The *Maker Hand*, *SoftHand Pro*, *ReHab TECH* and *Imperial ARM* exhibit many fingers inter-connected for a more adaptable solution of their grasp while keeping features from dexterous hands [18].

**Fig 2:**
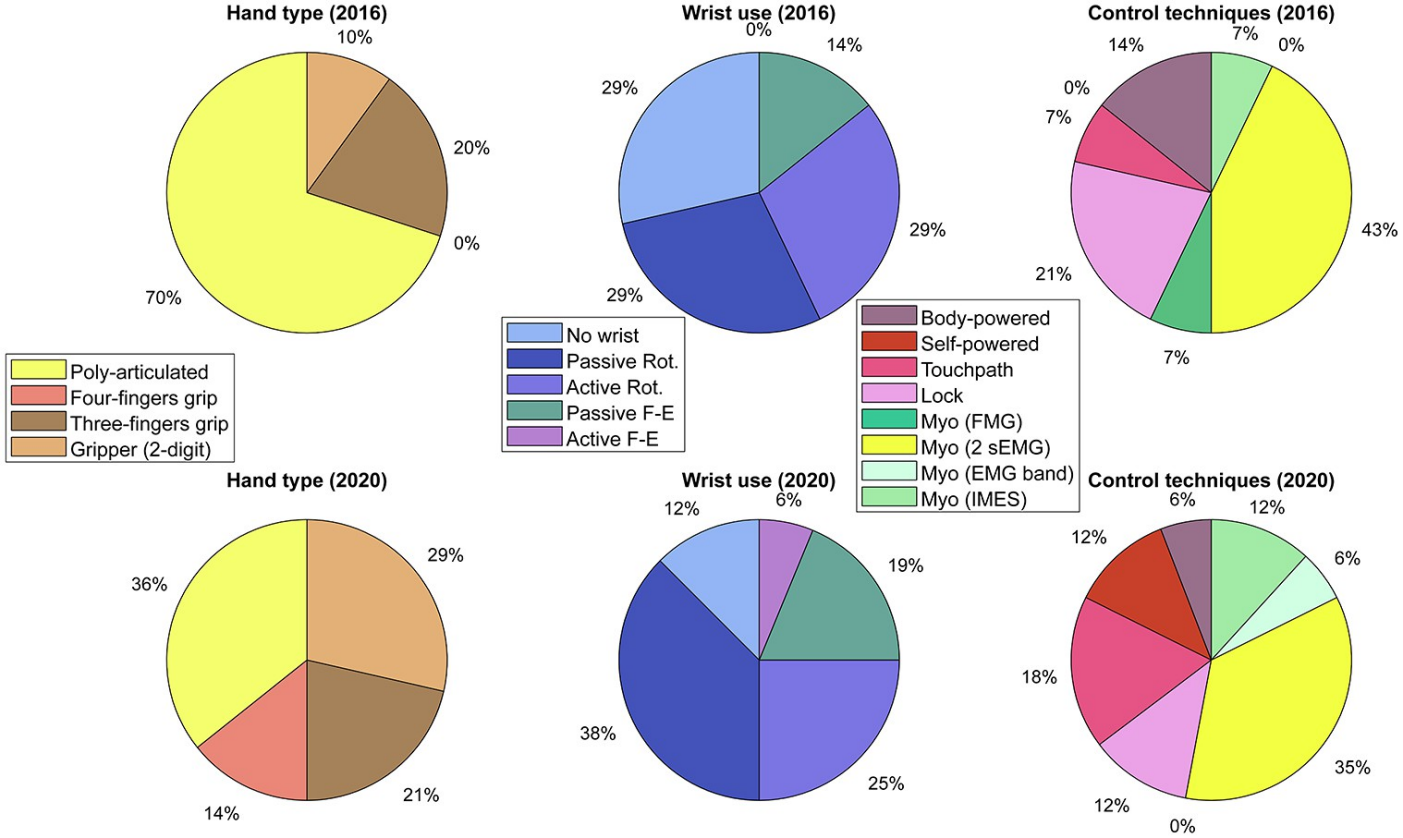
Overview of the participation between Cybathlon 2016 and 2020. The plots provide indications regarding hand type, wrist use and control techniques adopted in both editions.

As reported in Fig 1, many participants combine different methods and solutions for each joint (i.e. including passive thumbs or having different combinations of active and passive DoF at the wrist level). Comparing the contribution of the different technologies in both Cybathlon editions (see Fig 2), we observe a smaller participation of poly-articulated hands, from 70% to 36% in 2016 and 2020, respectively. We also observed higher participation of grippers, from 10% in 2016 to 29% in 2020. Regarding rotational wrists, in 2020 there has been an increase in passive solutions (29 to 38%) instead of active rotational joints (29 to 25%). For passive flexion-extension wrists, results suggest that in 2020 there is a larger appreciation of this DoF (14 to 25%). Note that 6% of teams even included an active flexion-extension wrist. Finally, in terms of control techniques, the body-powered represents a smaller percentage compared to the first edition, from 14% to 6%. While myoelectric hands in 2016 represent a 57% of the participation, in 2020 there is a slightly smaller participation (53%). However, among them, it is possible to find solutions that are emerging in the market, such as multiple EMG sensors embedded in a cuff. The latter is getting interest in literature for robustness and multi-DoFs control through machine learning techniques. Note that for self-powered systems, there is a lower percentage of locking options (21 to 12%) and a higher of touchpads (7 to 18%) to control closures or select among multiple grips or active joints.

### 4.2 Functional assessment with ACMC

#### 4.2.1 Method validation

**Fig 3:**
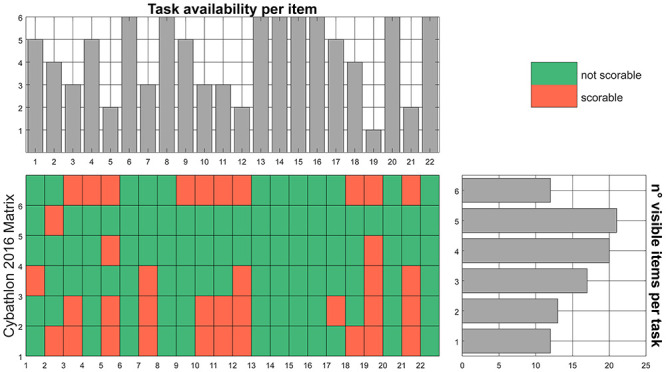
Evaluation of the ACMC for the race of Cybathlon 2016. In the central area, the matrix of the number of tasks per ACMC item is presented. There, red means that no scoring is possible (=0), while green proves its possible observation (=1). In the upper part, the number of tasks in which an item can be observed, and thus scored, is present. In the right side, the number of items that may be observed per task. The largest, the more complete is a defined task, and closer to real at-home conditions.

**Fig 4:**
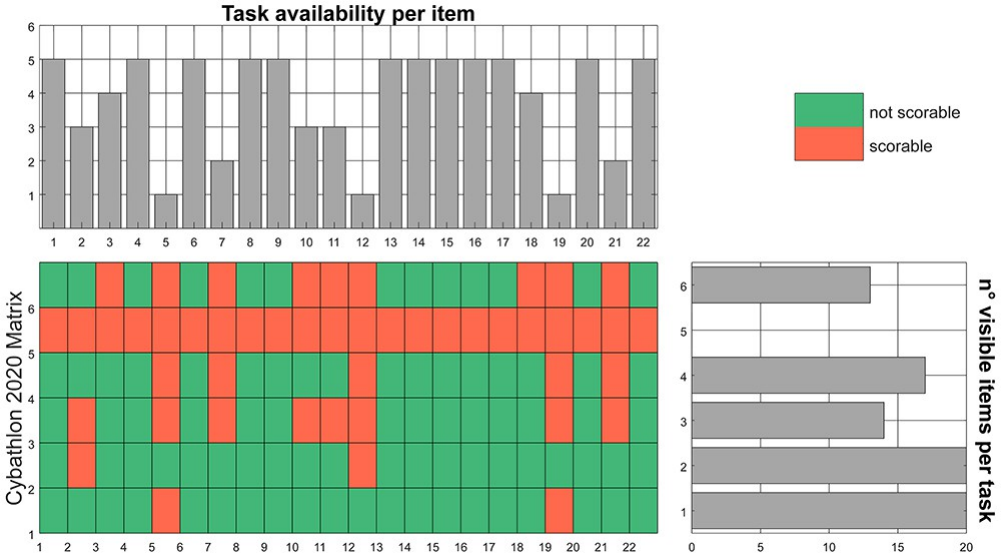
Evaluation of the ACMC for the race of Cybathlon 2020. In the central area, the matrix of the number of tasks per ACMC item is presented. There, red means that no scoring is possible (=0), while green proves its possible observation (=1). In the upper part, the number of tasks in which an item can be observed, and thus scored, is present. In the right side, the number of items that may be observed per task. The largest, the more complete is a defined task, and closer to real at-home conditions.

First of all, we validate the use of the Cybathlon race as a possible “scenario” for the ACMC. Most of the activities proposed in the race are inspired by ADL and the proposed analysis evaluates which ACMC items are observable in each Cybathlon task, reporting 1 if observable or 0 otherwise. The resulting matrix, reported in and Figs 3 and 4, proves the use of Cybathlon as a platform to score the ACMC. In Cybathlon 2016, Fig 3—***Task availability per item***, one item (*19—releasing in different positions*) is observable only in one task. Three items (*5—grasping in different positions*, *12—holding with support*, *21—coordinating both arms when releasing*) may be scored in only two tasks. In Cybathlon 2020 (see Fig 4—***Task availability per item***), three items (*5—grasping in different positions*, *12—holding with support*, *19—releasing in different positions*) are only visible in one task. Fig 4—***n° visible items per task*** shows that task #5 (haptic box) do not allow the evaluation of any item inside of the ACMC measure since the potential grasping is hidden from the observers. In both editions, the breakfast (task #4 in 2016 and #1 in 2020) and clothes (task #5 and #2 in 2020) present the highest number of items observable (>20) giving their affinity with real-life situations and applications. Moreover, these tasks permit the use of both hands, which feels natural for participants.

#### 4.2.2 ACMC score

**Fig 5:**
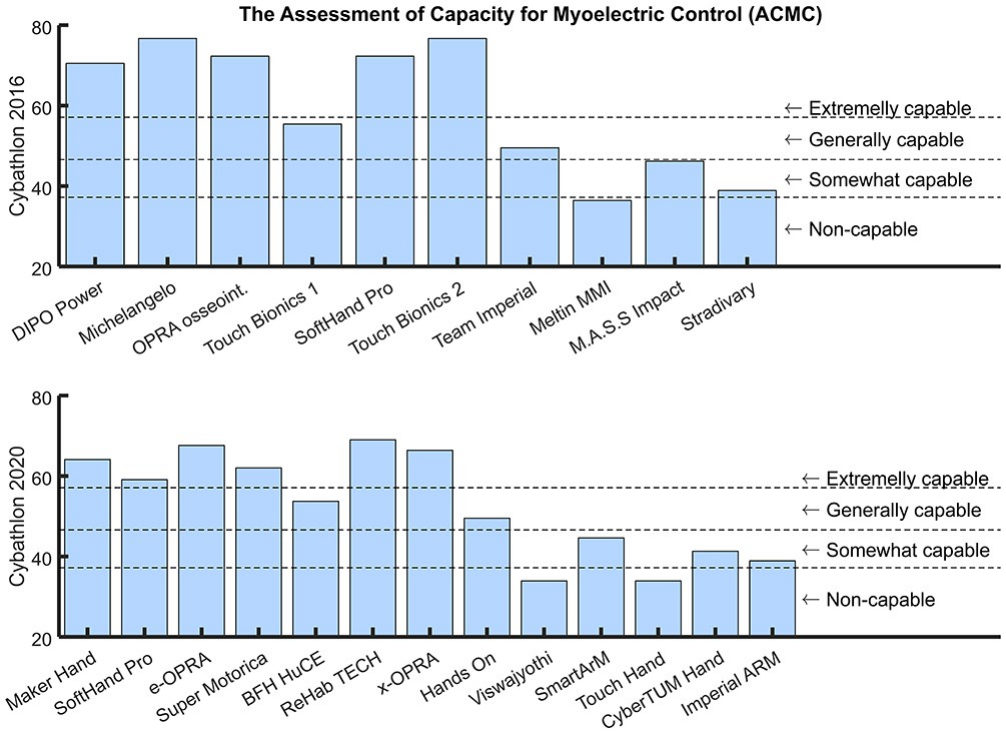
ACMC scoring for Cybathlon 2016 and 2020. The order of the proposed technology by teams is the result in each race, from right to left. The ACMC score goes from 0 to 100 points. The highest score, the more capable is considered the subject or technology under evaluation of its use at-home conditions. On the right side, the clinical condition defined by the assessment is reported.

Fig 5 presents the ACMC score for all participants (in race classification order) for both Cybathlon editions. Due to the limited sample of prostheses, it is difficult to perform a statistical analysis to this metric. In addition, this is especially hard in this context due to the different technological factors that interact among teams and has an influence on the final ACMC score obtained. For this reason, the authors considered it appropriate to base our results on the clinical scale evaluation and MDC, already proved valid to distinguish technologies in [15]. In 2016, there is a clear difference between the market and/or advanced systems (clinically considered *extremely capable*) and those that are still in a low Technology Readiness Level (TRL), which can be related to both the hardware and the software proposed. Among commercial prostheses, the more advanced self-powered rigid hands, e.g. team *Michelangelo* or *Touch Bionics*, obtained the highest ACMC score. Note that this is not the case of *Touch Bionics 1*, which participate with the i-limb in combination with an active rotational wrist, and enabling multiple grasp patterns through switching control techniques. A similar behavior occurs for participants of Cybathlon 2020, where the six systems that scored as *extremely capable* present a more finalized design, such as *OPRA* or *ReHabTECH*. Two teams with reliable but simple grippers, such as *BFH HuCE* and *Hands On*, that resulted *generally capable*. More prototyping solutions or systems that involved the control of multiple joints obtained lower ACMC scores. Only teams *Viswajyothi* and *Touch Hands*, which competed with a prototype designed for developing countries, obtain the score of *non-capable* in the clinical evaluation.

#### 4.2.3 Detailed analysis of ACMC item scoring

**Fig 6:**
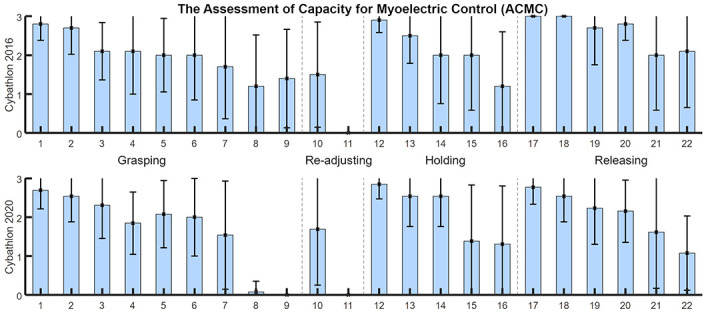
ACMC average item score (± SD) for Cybathlon 2016 and 2020 participants. The ACMC item score goes from 0 to 3. The highest score, the more capable is considered the subject or technology under evaluation of its use at-home. The items are grouped in 4 different manipulation actions of interest: grasping, re-adjusting, holding and releasing, separated by vertical dashed lines.

Fig 6 shows the average ± SD for each ACMC item among participants of each Cybathlon event. For each item group (i.e. Grasping, Re-adjusting, Holding, Releasing), we observed that low scoring is mostly related to manipulation actions *without visual feedback*, i.e. items 8, 9, 11, 15, 16 and 22. This might be induced by the stress of the competition, while the use of the prosthesis without visual focus on the hand can be encouraged in more relaxed situations. Note that the SD is larger for items 7, 21 and 10, which refer to the *coordination of both hands* and *repetitive grasp and release*, respectively, items that are especially interesting for natural and at-home use of prostheses.

**Fig 7:**
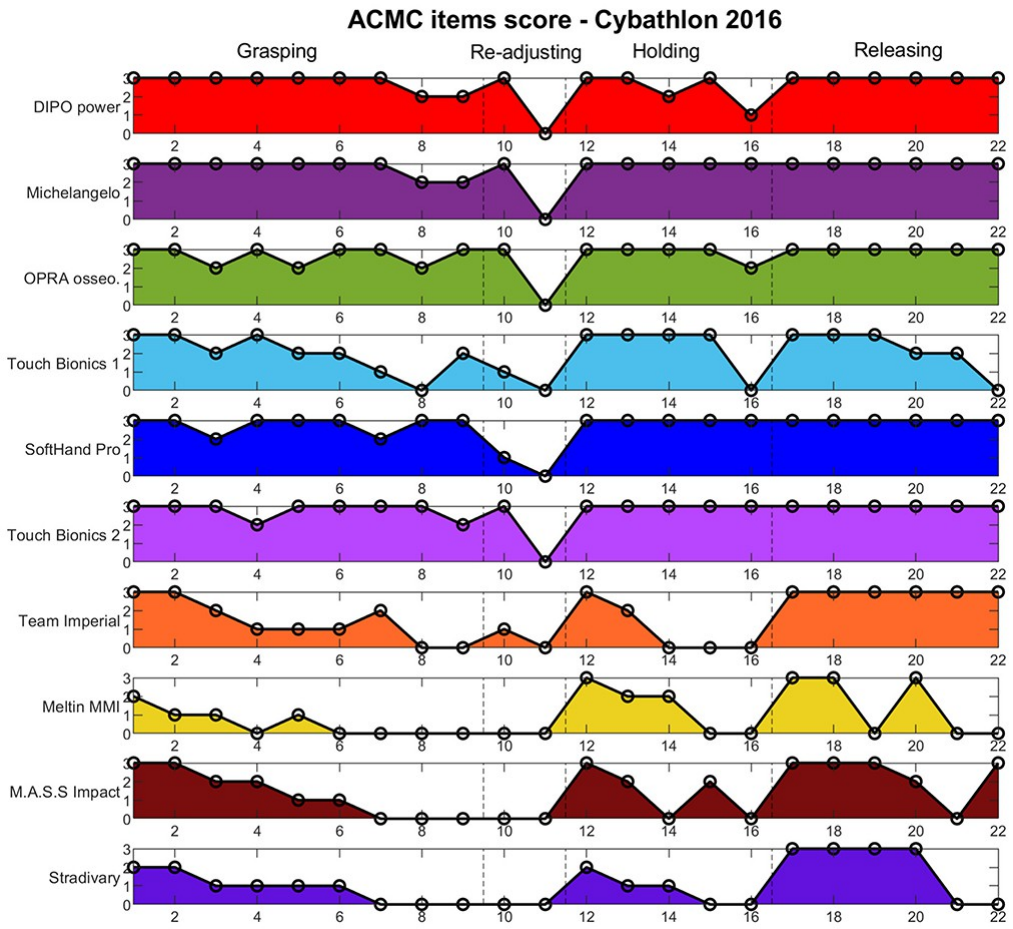
ACMC items score for Cybathlon 2016 participants. Each ACMC item (x axis) receives a score that goes from 0 to 3 (y axis). Subjects or technologies that obtain higher scores should present more proficient at-home use. The items are grouped in 4 different manipulation actions of interest: grasping, re-adjusting, holding and releasing, separated by vertical dashed lines. Participants are presented vertically in the classification order.

**Fig 8:**
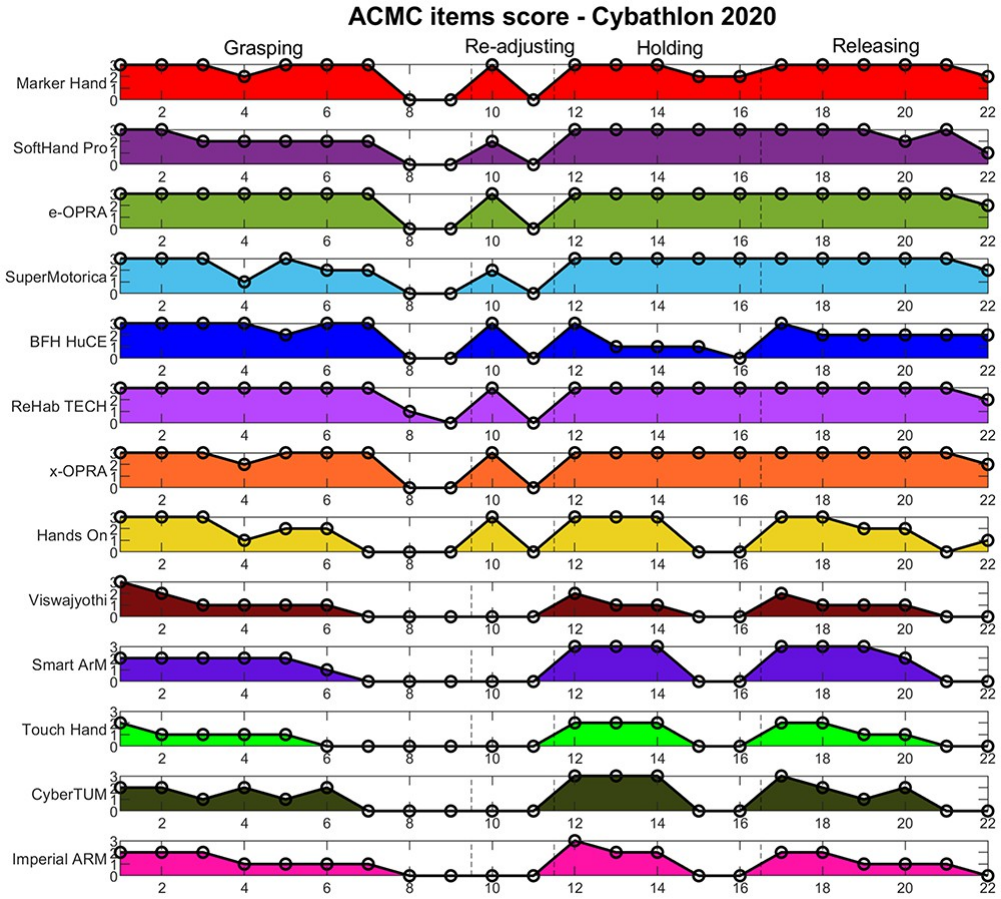
ACMC items score for Cybathlon 2020 participants. Each ACMC item (x axis) receives a score goes from 0 to 3 (y axis). Subjects or technologies that obtain higher scores should present more proficient at-home use. The items are grouped in 4 different manipulation actions of interest: grasping, re-adjusting, holding and releasing, separated by vertical dashed lines. Participants are presented vertically in the classification order.

Figs 7 and 8 show the raw score of each item for all participant technologies. In 2016, technologies considered *extremely capable* of being used at home (see Fig 5) showed especially high and stable scoring at holding and releasing phase items, with certain variations on the grasping action. Technologies with lower ACMC scores showed slightly lower grasping abilities, but larger variations in the holding and releasing items, which disfavors a comfortable use of an assistive device at home and without external help. Similar results are observed for 2020 (Fig 8), where *extremely capable* technologies present stable use mostly during holding and release phases. Among them, hands at the prototype level showed lower scores regarding grasping capabilities.*Generally capable* technologies present a significant decrease in performance regarding their ability to support users during holding or releasing phases (e.g. *BFH HuCE* and *Hands On*). The rest of the teams obtained a low ACMC score (from *somewhat capable* to *not capable*) in all manipulation phases.

### 4.3 Results from Cybathlon 2016 race

#### 4.3.1 Task-related

S1 Fig presents the *average time* among all participants (when succeeding) and the *best time* obtained (by a pilot) in each task. A large difference between *best time* and *average time* suggests less consistency among the technological solutions that participated, indicating which aspect (or task) research must focus on. The task with the highest lack of consistency in absolute value corresponds to #3, which evaluates the ability to grasp and manipulate in different positions and body configurations. In percentage, tasks #1 and #6 present larger discrepancies among systems, which relate to difficulties in grasping objects of different sizes and bilateral coordination. The percentage of occasions in which pilots fail in performing a task, including both the attempt with failure or not even attempting a task (marked as X), gives an indication of the level of difficulty of the task. Results highlight a strong difficulty in performing task #2 with 70% of failures.

#### 4.3.2 Technology comparison

**Fig 9:**
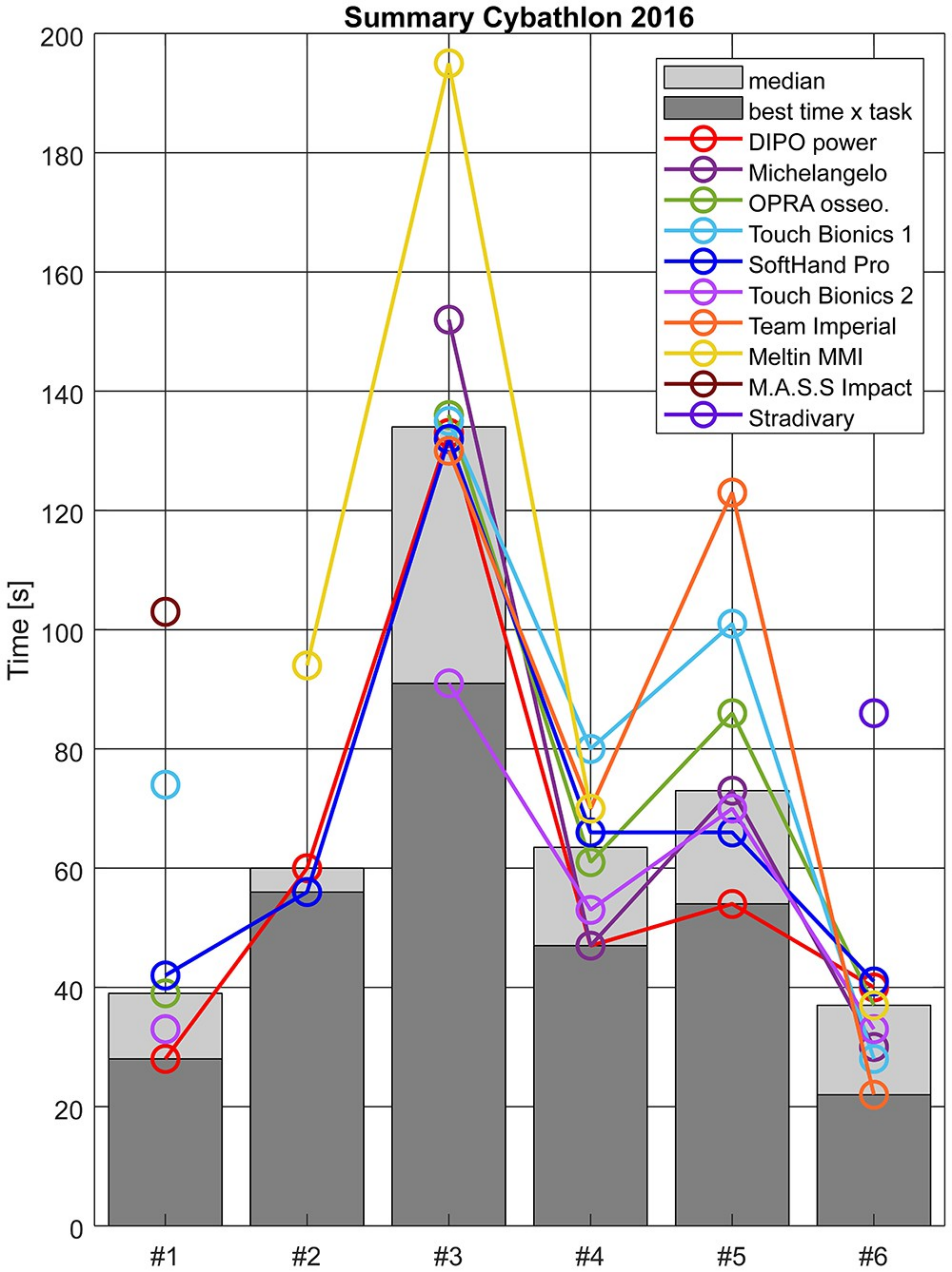
Detailed results from participants at each task during Cyabthlon 2016. Note that the colored lines for each team helps the visualization of the marker of each team corresponding to the performance of each task, and do not represent a tendency among tasks.

For a more precise description of the advantages observed among technologies, Fig 9 shows a summary of the results of Cybathlon 2016, including the time performance per team. Task #2 evaluates the ability to continuously orient the end-effector and resulted the hardest. This action directly addresses issues at the wrist joint, both in design and control solutions. However, none of the participants with an *A RW* complete the task successfully in the finals and two of those that were able to complete it were body-powered systems (i.e. no wrist joint). In task #3, *Touch Bionics 2* outperformed the rest of the teams. They used a multi-DoF rigid hand (equal to *Touch Bionics 1*), but a simpler control strategy was used with only grasp pattern and a passive thumb rotation. Results for tasks #4 and #6 are pretty consistent among technologies, In task #5, both *Team Imperial* and *Touch bionics 1* are the slowest, even though competing with an advanced multi-grip hand (grip selection through switching techniques) and an active rotational wrist.

### 4.4 Results from Cybathlon 2020 race

#### 4.4.1 Task-related

S2 Fig presents the *average time* among all participants (when succeeding and including the three repetitions) and the *best time* obtained in the task by a pilot. A larger difference between *best time* and *average time* suggests less consistency between systems. These results give an overview of the design requirements that must be further addressed and improved in a future generation of prostheses. During this event, the tasks with a larger lack of consistency (both in absolute value and percentage) correspond to #2 and #3, which mainly focus on ADL and precise grasping, safe holding and releasing. The percentage of failures (including both the attempt with failure and not even attempting a task) allows us to validate which is the hardest task to complete and if these difficulties correspond to the points assigned for its completion. The results match the level of difficulty (associated points), except for task #1. Note that tasks #5 and #6, which include components that are less commercially-available (i.e. sensory feedback and wrist rotation), obtained failures for more than the 50% of the races.

**Fig 10:**
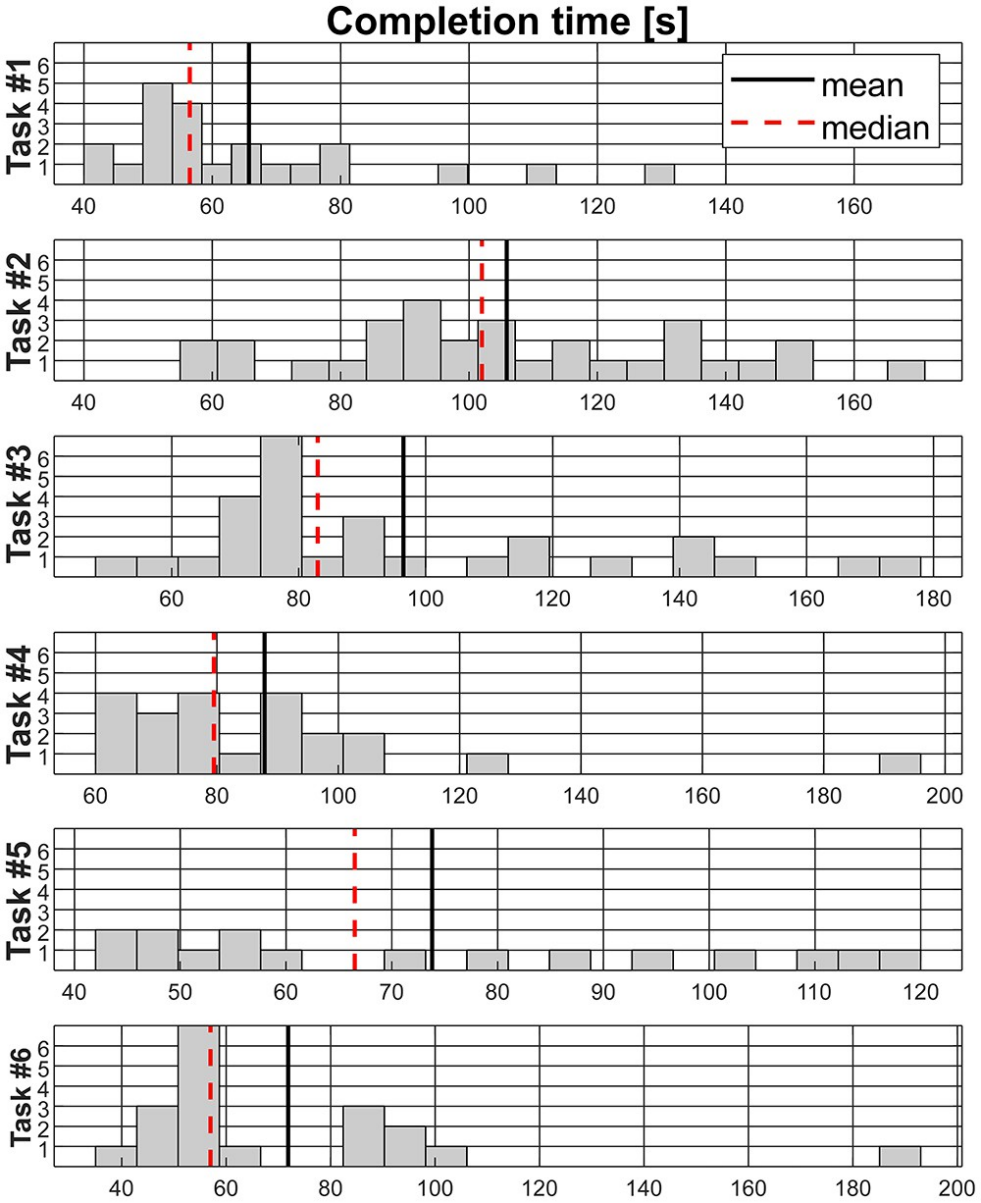
Histograms of the time executed per task among participants.

Fig 10 presents the data distribution for the task execution time (including all successful cases). The less consistency among results suggests a higher variability in performing such tasks by the participant technologies. Tasks #1, #4 and #6 showed consistent timing among pilots. More divergent results are observed for tasks #2, #3 and #5. The latter focuses on sensory feedback for shape and material discrimination, which has no standard and accepted solution in the market and is a topic still under exploration in research. A detailed analysis of tasks in which technologies are divergent represents an interesting context in which the features that favor a more complete and competent prosthetic solution can be evaluated.

**Fig 11:**
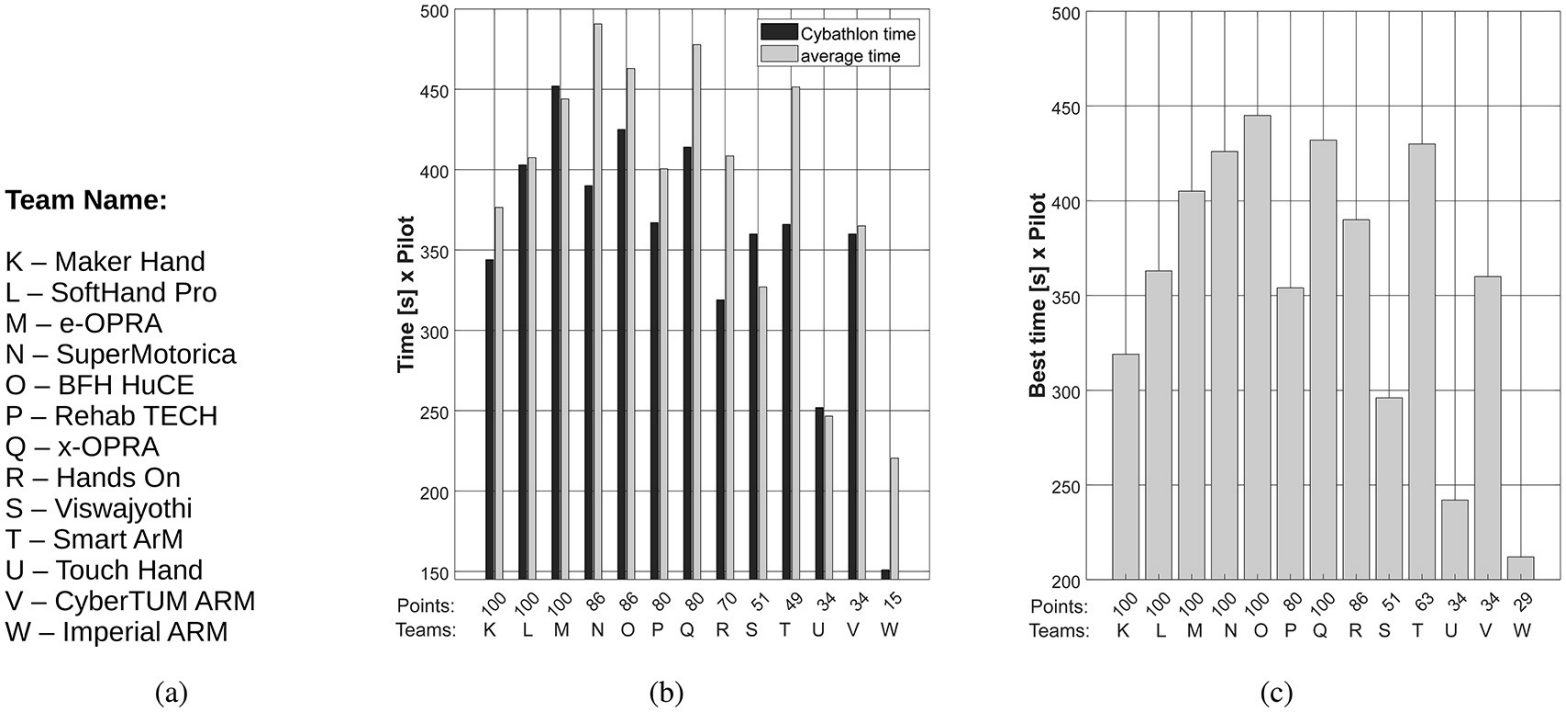
Pilot performance. (a) reports the team name, (b) shows the *Cybathlon time* and the average time (n = 3 races) per pilot and (c) presents results from a hypothetical race per pilot (using their best time per task among repetitions).

Additionally, Fig 11(B) presents data for each pilot ordered by its corresponding rank. Data includes the *average time* among the three repetitions and the time used for Cybathlon as the final race (i.e. *Cybathlon time* as the team best race). For the *average time*, we consider all the times that the pilot has successfully done the tasks during the 3 trials. The x axis shows the received points by each pilot during their best race. The smaller the difference between *Cybathlon time* and *average time*, the more consistent is the use and reliable is the technology. Note that the smallest difference is obtained by teams L, M, U and V. To mitigate the influence of the competitive environment and highlight the device capabilities, we analyzed a hypothetical race, shown in Fig 11(C). We consider the best time per task done by each pilot among the three repetitions and calculate the total points that the pilot could hypothetically receive (x axis). While in the real competition, only 3/13 participants were able to execute all tasks and get 100 points, in the hypothetical competition, 6/13 participants are capable of doing correctly all tasks at least once.

#### 4.4.2 Technology comparison

**Fig 12:**
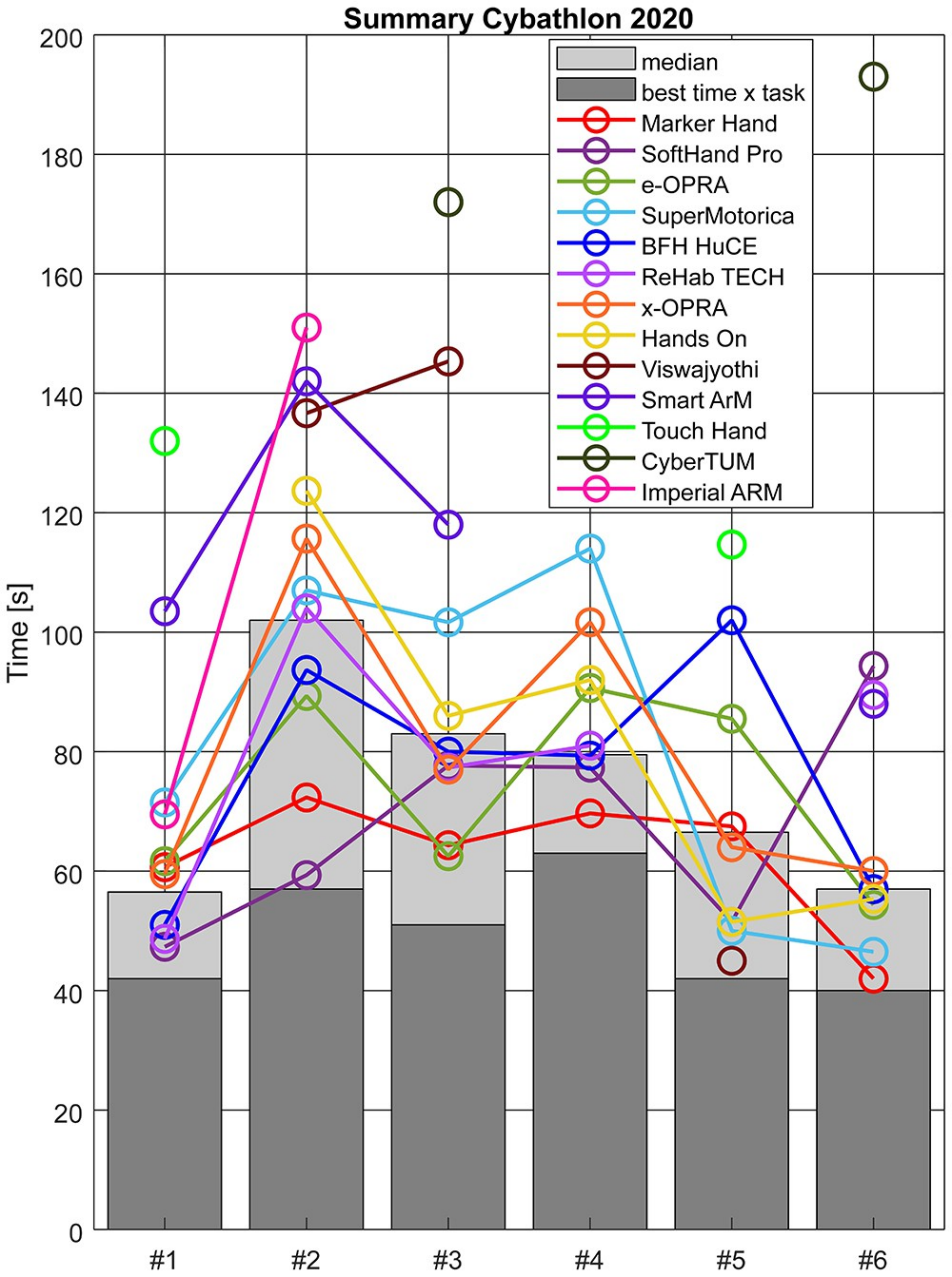
Detailed results from participants at each task during Cybathlon 2020. Note that the colored lines for each team helps the visualization of the marker of each team corresponding to the performance of each task, and do not represent a tendency among tasks.

For a more detailed description of the advantages observed among technologies, Fig 12 reports a summary of the results of Cybathlon 2020, including the average time performance per team (including the three races by each team). Regarding task #1, only the *SoftHand Pro*, *ReHab TECH* and *BFH HuCE* hands showed a higher performance than the median. While the *Imperial ARM* and *SuperMotorica* needed +15s with respect to the median, the *Smart ArM* shows a worse performance (about +50s). Finally, the *Touch Hand* had the worst performance.*Viswajyothi*, *Hands On* and *CyberTUM* failed (X) at executing this task, probably due to still a bulky or preliminary design phase of the system. In task #2, both the *Touch Hand* and *CyberTUM* did not participate (X). The other technologies present a big dispersion. For task #3, only the *Touch Hand* and *Imperial ARM* are not present, even though both of them tried. Note that this task includes many and diverse objects, but these bulky hands still present a research prototype design version. Accordingly, *CyberTUM* and *Viswajyothi* performed the worst. Results from task #4 reported *Maker Hand*, *SoftHand Pro*, *BFH HuCE* and *ReHab TECH* as the most robust systems. Note that the last five in the ranking (which are more of a prototype version, except for the i-limb of *Smart ArM*) were not capable of completing the task. The *BFH HuCE*, *e-OPRA* and *Imperial ARM* are the only teams including sensory feedback, but their results in task #5 were not excelling compared with other groups. Finally, task #6 focuses on the use of the wrist and safe holding. The level of amputation at the transradial level plays an important role in the user’s range of motion at pro-supination. Among participants, the *Maker Hand* and *Touch Hand* do not present any (passive or active) DoF at the wrist level. While the *Touch Hand* did not participate, the *Maker Hand* was the best performer. Active rotational wrists were tested by *Imperial ARM* (not capable), *Smart ArM* and *Hands On*. However, they did not obtain significantly better results compared to other solutions.

## 5. Discussion

The performance of upper limb prostheses is influenced by various factors. Although some of these aspects are presented separately in this section for a more detailed examination of each aspect, it is important to acknowledge that they often intersect and influence one another. By examining each aspect individually, we highlight their significance in achieving effective artificial limbs. However, understanding the interplay between these factors provides valuable insights into the complex nature of prosthetic performance, empowering researchers and developers to make informed decisions aimed at enhancing overall functionality.

### 5.1 Results of the ACMC score

(Fig 5) show a notable difference between market-oriented systems and research prototypes. In both editions, 50% of the participants scored as *extremely capable*. Among them, advanced self-powered commercially-available rigid hands obtain the highest ACMC scores, indicating high capacity and ability to be used for activities of daily living and at-home conditions. On the contrary, prostheses with a lower TRL present slightly lower ACMC scores. However, prostheses that showed potential for home use, do not necessarily perform best in the Cybathlon race condition, where strict time limits and specific rules could strongly influence pilots’ performance. For instance, in 2020 only pilots L and M showed consistent results among the three trials (Fig 11(B)) and are capable of performing all the tasks (i.e. 100 points), suggesting high expertise. Nonetheless, the analysis of the hypothetical race (Fig 11(C)), shows that 6/13 participants are capable of executing correctly all tasks at least once (among all trials) and get 100 points.

When looking at the ACMC raw score (0–3) given to the pilots’ performance on each ACMC item, the areas in which the specific features of novel technologies demonstrate problems are shown in and Figs 7 and 8, rather than the prosthesis level of readiness for real-life scenarios. An additional relevant aspect is the device wearability and compactness, which affects also functionalities and usability. In 2020, this could have influenced the decision of some teams (e.g. *Touch Hand*, *CyberTUM*) to not even try task #2, which involves activities such as wearing a sweater.

### 5.2 ADL and bilateral coordination

Activities of Daily Living have inspired therapeutic training of assistive and rehabilitation devices for many years and provides a relevant indication of reliability in real-life environments. According to the ACMC results reported in and Figs 3 and 4, breakfast and clothes tasks in both editions represent strong similarity to real scenarios and the closest condition to at-home use of prostheses. While the development or assessment of artificial hands mostly focuses on grasping actions, the different phases of manipulation, such as holding and releasing in ADL, should be more investigated. For instance, S2 Fig shows that tasks #2 and #3 from the 2020 race present a larger discrepancy between *best time* and *average time* to complete them. Moreover, these tasks include small objects that require precise grasping. In agreement, Fig 10 highlights that a more divergent histogram occurred for tasks #2, #3 and #5. The large variability in task execution suggests that only some technologies might be suitable for ADL and dexterous grasping requirements.

Unilateral amputee subjects usually tend to execute several tasks as one-handed actions, entrusting the prosthesis mostly with a supportive role. However, a more active role of artificial limbs is necessary for bimanual actions, which provides an important indication of the translation of technology in real life. Two ACMC items (7—*coordinating both arms when grasping*, 21—*coordinating both arms when releasing*) evaluate the quality and level of coordination between the artificial and intact arms. Unfortunately, in 2020, these items can be scored only in two tasks (tasks #1 and #2, see Fig 4), which represents less than 50% of the race. Nonetheless, the results of S2 Fig indicate that the percentage of failures matches with the level of difficulty (i.e. associated points), except for task #1. While task #1 allows the use of both hands (commonly one intact hand and one prosthesis) for most of the actions, it has a similar failure percentage to task #4, which is considered one of the hardest activities of the competition due to the required fine manipulation skills. These observations highlight the consideration of bimanual coordination as a fundamental aspect to evaluate commercially-available solutions and to push forward more adaptable solutions.

### 5.3 Mechatronics and dexterous grasping

Fig 12 shows that hands with rigid properties (e.g. *Maker Hand*, *e-OPRA*) were especially good in dexterous grasping (task #3) as they can provide users with more **precise** or repetitive **grasps**.*Maker Hand*, the winner of the Cybathlon 2020 edition, uses a 3D printed hand with a fully mechanical actuation and anthropomorphic shape. A similar approach was successful also in 2016, where *DIPO Power* used a hook-like shape. The second-best performance for task #3 includes solutions with a more minimalist design approach, such as under-actuated systems. While their less precise or repetitive grasps could have influenced in negative results for task #6, their adaptability accounts for their use in multiple and variable conditions. For instance, the potential of combining *soft robotics* and under-actuation is highlighted in the results of Fig 12, where team *SoftHand Pro* outperforms in both tasks #1 and #2, strongly related to ADL.

Note that team *BFH HuCE* is the only team that participated in 2020 with two different mechatronics solutions, a research gripper and a customized version of a commercial hand. The latter is a myoelectric hand (MyoHand VariPlus Speed, from Ottobock), from which little and ring fingers were removed. These fingers were merely aesthetics and could occlude the visibility while grasping. In task #3, this team decided to use the research gripper (2-digit), which embeds a lock feature (see Fig 2). Therefore, this choice may be strongly related to the unreliable holding phase with the commercial hand, which can be solved with the inclusion of a mechanical lock for some prosthesis users.

Among the different manipulation phases considered, ACMC items related to holding and releasing generally present lower values even though these are necessary for skillful technologies (see and Figs 7 and 8). Pure body-powered solutions (e.g. *DIPO power* and *Maker Hand*—who won both cybathlon race editions), showed certain limitations in the **holding** phase compared to standard myoelectric hands, but proficient scoring at grasping items. Unlike self-powered options, body-powered solutions do not require continuous active control once an object is grasped. This could significantly improve the velocity in holding and turning, especially in task #6. Indeed, the *Maker hand* performed the fastest in this activity (see Fig 12).

Despite the importance of the wrist joint in ADL and to limit compensatory movements, less attention is given to the mechanical design of active wrists. In both editions, more than 50% of the participants do not use a wrist or prefer to include a passive solution for pro-supination or flexion-extension. Among the few teams that used an active wrist, it is possible to observe the use of active solutions for wrist FE in 2020, an option that is still not available in the market.

**Robustness** represents a fundamental feature for systems in ADL or hobbies, as highlighted in task #4. Fig 12 showed that three teams did not even participate in this task. Among them, the *Smart ArM* includes a commercially-available poly-articulated hand with rigid properties. This may be related to the amputation level (even though it is equal to the osseointegration teams), or to the fragility of the i-limb. Also, note that the participants with osseointegration performed slightly worse in this task compared to the average. This might be a consequence of vibrations transmitted directly to skeletal segments or to the distal level of amputation.

### 5.4 Control modality and control of multi-DoFs

In both Cybathlon editions, a simple body-powered solution outperformed more advanced systems from research and market. Body-powered systems only control one of the directions of actuation (e.g. normally closed; only commands the release of objects), which can be more convenient in terms of physical and mental load, and reliable for a competitive environment. Among myoelectric devices, standard switching control techniques to select among **multi-grip** or an active rotational wrist were the preferred option by some participants, e.g. *Team Imperial*, *Touch bionics 1*. Despite this strategy being available in commercial devices, they performed the slowest in the 2016 race at task #5 (see Fig 9), which suggests that this method may be excessively complex for fast and natural execution. In accordance, advanced commercially-available hands, such as the i-limb used by *Touch Bionics 1* and *Smart ArM* obtained low ACMC scores (see Fig 5), highlighting the lack of control **intuitiveness** or multi-DoF coordination of current solutions. For instance, teams showed an increasing interest towards passive rotational wrists (+9% in 2020—reported in Fig 2). A reason might be related to the limitations of myoelectric control methods for *hand + wrist* coordination, which is still a challenge faced by many research groups. This is also highlighted by the performance of *CyberTUM* and the *Smart ArM* in task #6. Despite these teams used active F-E wrist and active R wrist, respectively, they performed the worst. Among teams implementing active rotational wrist in 2020, *Hands On* performed the best in task #6 (see Fig 12), as it presents a specific modality to turn 180° without the need for continuous and precise control of its orientation. The combination of multi-DoF hands or arms with novel and robust control strategies, i.e. using machine learning or shared-control techniques, could represent an interesting alternative.

Apart from robust intention detection and intuitiveness of control methods, the control reliability in different limb positions for grasping and releasing is a fundamental component for the successful execution of ADL. This issue is highlighted in S1 Fig, where task #3 of Cybathlon 2016, which focuses on this aspect, presents large variability among systems. S1 Fig shows that task #3 presents less consistency among systems during Cybathlon 2016. This activity relates to the execution of **compensatory movements** due to a lack of dexterity of the arm and control robustness when moving the arm in space. Both these issues are topics of interest in recent literature with novel share-control methods or physical solutions to increase the users’ workspace. For instance, some teams show interest in including passive or active flexion-extension wrists. Nonetheless, S1 Fig shows that in 70% of the occasions, the technologies fail at completing task #2, which strongly relates to compensatory movements and lack of wrist orientation and control. Accordingly, an active rotational wrist (*A RW*) could help in performing this task. However, the smoothness and precise control required is not yet available among participants. Results Fig 9 from show that none of the participants with an *A RW* would complete the task successfully in the finals, contrary to the two body-powered solutions that were able.

### 5.5 Exploration of sensory feedback

Despite none of the teams from Cybathlon 2016 implemented a haptic feedback mechanism, at least three teams (*BFH HuCE*, *e-OPRA*, *Imperial ARM*) in 2020 included a strategy to restore sensory information, which is especially useful for task #5. The research device of *BFH HuCE* and *Imperial ARM* provide feedback through an external device in contact with the skin, but *e-OPRA* uses implanted sensors. Unlike *Imperial ARM*, the other two groups (*BFH HuCE* and *e-OPRA*) were able to successfully complete the task but with larger times than other participants without sensory feedback. This may occur because pilots invest a lot of time to understand the features or because the type of feedback given was not the most appropriate for the task. In some cases, the type of sensation delivered is related to the grasping force and not to the identification of the object’s shape. This may explain the similarities between groups using or not sensory feedback. Note that some of the most simple and rigid hands (*Viswajyothi*, *Hands On* and *SuperMotorica*) performed well in task #5, probably due to the vibrations perceived through the physical interface. The previous results and the corresponding literature highlight the need for studies evaluating the effectiveness of haptic devices in real-life situations [39]. Indeed, one area that still struggles with clinical translation is completing the control loop itself through understandable and sufficient sensory feedback.

## 6. Study limitations

During the ACMC evaluation, the expert observer focused on the features of the technology and how they affect the capacity of the pilot in performing several actions. The ACMC score is an interface between a person and technology; if components and/or the person’s ability improves, the ACMC score should entail it. Nonetheless, it is difficult to separate the features of the technology from the inter-pilot capacities and their training hours when comparing solutions in different pilots. The authors of this survey considered the methodology appropriate as all participants had opportunities to train with their devices and arrive at the race with an adequate level of performance considered by their team members. Note that the ACMC is validated for myoelectric control and not for BP. The examiners used this outcome for BP control according to this limitation. For BP technology—voluntary opening (passive closing), the holding action is passive. However, if pilots present a low ability to control, the hand can open involuntarily. Therefore, good/excellent users can score high on holding even though it is a passive action. People who actively use their grip provides a greater basis for assessment, but also for failures, i.e. an extremely capable user is at risk of scoring lower than a somewhat capable user. The Cybathlon race accepts the passive use of prostheses (as support) and the use of pilots’ mouths or arm-/elbow pits to hold objects instead of using the terminal device. Another limitation occurs when scoring appropriate grip force w/o visual feedback. As many objects are solid and are not affected by applying high grip forces, the examiners could only score based on what is visible when/if the pilot demonstrates the item. For example, some participants make an excessive force in task #6 (Cybathlon 2020) while turning the cups. Using lighter plastic glasses/cups may help to evaluate not only wrist use, but an appropriate grip force. Moreover, note that the use of visual feedback differs between small and large objects, i.e. it is easier to grasp/release w/o visual feedback when grasping large objects. For instance, when gripping without visual feedback, small objects requiring precision are often looked at whereas large objects that require less precision (e.g. clothes-hanger) require less visual feedback. Note that regarding the release without visual feedback, the examiners do not judge the ability to let go of the object, but the ability to let go without looking. The ACMC scoring in task #5 (Cybathlon 2020) for sensory feedback was not possible, but no specific improvement from any of the technologies that present sensory feedback is observed in completing the task (see Fig 12). Different levels of amputation for transradial pilots, which correspond to the length of the stump can affect a lot the capacity for pro-supination of the pilot (visible in pilots performance during task #6 (Cybathlon 2020)). Something that requires the rotation of the wrist while using also the counterpart would favor the use of a natural body posture for the coordination of both movements and the evaluation of the wrist joint.

On a more technical note, during the evaluation of the ACMC scoring by an expert, some issues came out. Re-adjusting grasp is only visible if the pilot fails for some blank tasks (except for haptic, in which the grasp is not visible). There is no specific object included in the task that requires re-adjusting grasps, as it could be the folding of a tablecloth. Even though results in Figs 3 and 4 and showed no specific inconvenient in the evaluation of visual feedback in Cybathlon, both the quality of video recordings and the race condition may hinder the scoring of the related items, as observed in Fig 6. Indeed, the videos of 2020 may be difficult to score as some aspects are not visible in detail. For example, there are usually difficulties in the visual feedback scoring and sometimes the videos do not provide a precise vision of the use of the prosthesis. Furthermore, grasping in different positions is only observable in tasks where more than 2 locations are included, which is an uncommon situation during the race setup.

## 7. Conclusions

This work assesses the functionality of many state-of-the-art upper limb prostheses, both commercially-available and research solutions, in the same conditions, provided by the Cybathlon race. For this analysis, we used the Assessment of Capacity for Myoelectric Control (ACMC). Adding variability to the assessments of novel assistive and rehabilitation technologies, as occurs in the ACMC, accounts for everyday situations where the exact circumstances of a given task are not always known and/or constant over time and across different locations.

With a critical perspective, the authors bring insights into both questions: (i) which trends may be beneficial in the future for more complete and satisfactory artificial limbs used at home and (ii) how to evaluate properly an assistive device. Despite some limitations, this is the first evaluation that offers a quantitative analysis of potential emerging trends in the field and emphasize the need for further research and development, with a particular focus on intuitive control of multiple DoFs, adaptive solutions and the integration of sensory feedback. Note that even assessments are continuously evolving to adapt to the external situation. Indeed, with the recent technological development of multi-grip prostheses, new items will be explored to propose an updated version of the ACMC assessment. These will include scoring the ability to select a grip or the use of an appropriate pre-grasping position of the arm.

## Supporting information

S1 Fig Powered-prostheses that participate in Cybathlon 2016. The last row of the table presents general results from Cybathlon 2016: the average and the best time, differences and the percentage of failures per task. Cells in green highlight that the task was successfully completed, while red cells represent tasks not completed (with 0 score and time achieved) or not even tried by teams (marked with X). The bottom rows show the maximum score (points) associated with the specific task (larger values indicate the more difficult or important tasks). (a-j) shows pictures of the technology used by the pilot in order of the final classification.
(PDF)

S2 Fig Powered-prostheses that participate in Cybathlon 2020 Global Edition. The last row of the table presents general results from Cybathlon 2020: the average and the best time, differences and the percentage of failures per task. Cells in green highlight that the task was successfully completed, while red cells represent tasks not completed (with 0 score and time achieved) or not even tried by teams (marked with X). The bottom rows show the maximum score (points) associated with the specific task (larger values indicate the more difficult or important tasks). (a-m) shows pictures of the technology used by the pilot in order of the final classification.
(PDF)

S1 Appendix (PDF)
