# Supplementary material for: Functional assessment of current upper limb prostheses: An integrated clinical and technological perspective
Source: PLoS One. 2023 Aug 16;18(8):e0289978. doi: 10.1371/journal.pone.0289978 (PMC10431634; doi:10.1371/journal.pone.0289978)
Supplement: S1 Fig — The last row of the table presents general results from Cybathlon 2016: the average and the best time, differences and the percentage of failures per task. Cells in green highlight that the task was successfully completed, while red cells represent tasks not completed (with 0 score and time achieved) or not even tried by teams (marked with X). The bottom rows show the maximum score (points) associated with the specific task (larger values indicate the more difficult or important tasks). (a-j) shows pictures of the technology used by the pilot in order of the final classification. (PDF) [file pone.0289978.s001.pdf]

Rank	Name	Fig.	Points	Time	Time						Team	Finals
1	A	12(a)	660	6:02	28	60	133	47	54	40	DIPO Power	A
2	B	12(b)	558	5:35	33	0	152	47	73	30	Michelangelo	A
3	C	12(c)	558	5:59	39	X	136	61	86	37	OPRA Osseoint.	A
4	D	12(d)	558	6:58	74	0	135	80	101	28	Touch Bionics	A
5	E	12(e)	660	6:43	42	56	132	66	66	41	SoftHand Pro	B
6	F	12(f)	558	4:40	33	0	91	53	70	33	Touch Bionics	B
7	G	12(g)	443	5:45	0	0	130	70	123	22	Team Imperial	B
8	H	12(h)	437	6:36	X	94	195	70	0	37	Meltin MMI	B
9	I	12(i)	115	1:43	103	0	0	0	X	0	M.A.S.S. Impact	C
10	J	12(j)	101	1:26	0	X	0	X	0	86	Stradivary	C
				**Tasks:**	#1	#2	#3	#4	#5	#6		
				**Points:**	115	102	130	104	108	101		
				**Average time:**	50.29	70	138	61.75	81.86	39.33		
				**SD time:**	27.78	20.88	28.77	11.95	23.53	18.53		
				**Best time:**	28	56	91	47	54	22		
				**Abs diff best vs. average time:**	22.29	14	47	14.75	27.86	17.33		
				**Diff best vs. average time [%]:**	44.32	20	34.06	23.89	34.03	44.07		
				**Fails [%]:**	30	70	20	20	30	10		

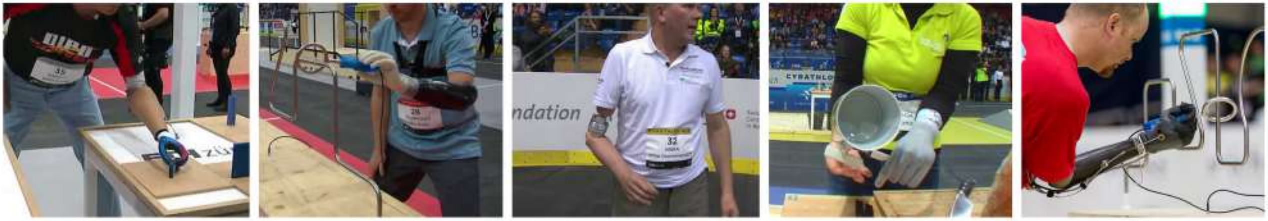

(a) (b) (c) (d) (e)

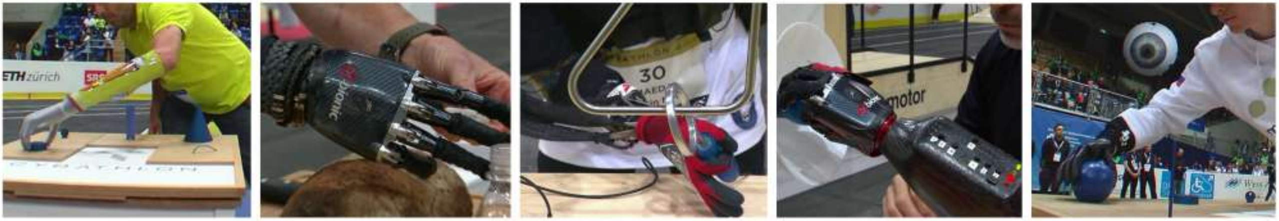

(f) (g) (h) (i) (j)
