# Supplementary material for: Functional assessment of current upper limb prostheses: An integrated clinical and technological perspective
Source: PLoS One. 2023 Aug 16;18(8):e0289978. doi: 10.1371/journal.pone.0289978 (PMC10431634; doi:10.1371/journal.pone.0289978)
Supplement: S2 Fig — The last row of the table presents general results from Cybathlon 2020: the average and the best time, differences and the percentage of failures per task. Cells in green highlight that the task was successfully completed, while red cells represent tasks not completed (with 0 score and time achieved) or not even tried by teams (marked with X). The bottom rows show the maximum score (points) associated with the specific task (larger values indicate the more difficult or important tasks). (a-m) shows pictures of the technology used by the pilot in order of the final classification. (PDF) [file pone.0289978.s002.pdf]

Rank	Name	Race rank	Fig.	Points	Total [min]	Time [s])						Team
1	K	1	13(a)	100	5:44	73	64	51	64	48	44	Maker Hand
2	L	2	13(b)	100	6:43	52	57	70	96	42	86	SoftHand Pro
3		-	-	100	6:46	48	58	73	66	61	100	SoftHand Pro
4		-	-	100	6:52	56	73	74	82	87	40	Maker Hand
5	M	3	13(c)	100	7:32	56	89	66	106	78	57	e-OPRA
6	N	4	13(d)	86	6:30	0	102	117	74	50	47	Super Motorica
7	O	5	13(e)	86	7:05	0	92	80	94	102	57	BFH HuCE
8		-	-	84	6:44	72	92	0	92	93	55	e-OPRA
9		-	-	80	5:28	57	87	59	74	0	51	e-OPRA
10		-	-	80	6:02	42	63	90	70	0	97	SoftHand Pro
11	P	6	13(f)	80	6:07	43	96	80	64	0	84	ReHab TECH
12		-	-	80	6:32	53	89	78	77	0	95	ReHab TECH
13		-	-	80	6:33	65	113	97	72	0	46	Super Motorica
14	Q	7	13(g)	80	6:54	56	95	77	126	0	60	x-OPRA
15	R	8	13(h)	70	5:19	0	124	0	92	47	56	Hands On
16		-	-	68	6:09	0	145	77	91	56	0	x-OPRA
17		-	-	66	5:30	63	107	0	88	72	0	x-OPRA
18		-	-	62	4:24	53	80	68	63	0	0	Maker Hand
19		-	-	62	5:00	51	92	87	70	0	0	BFH HuCE
20		-	-	62	5:53	50	127	74	102	0	0	ReHab TECH
21		-	-	62	7:51	78	106	91	196	0	0	Super Motorica
22		-	-	53	3:41	0	113	0	0	56	52	Hands On
23	S	9	13(i)	51	6:00	0	170	145	0	45	0	Viswajyothi
24	T	10	13(j)	49	6:06	0	152	126	X	X	88	Smart ArM
25		-	-	46	4:38	0	134	86	0	0	58	Hands On
26		-	-	48	4:04	0	97	73	74	0	0	BFH HuCE
27		-	-	45	5:44	97	138	109	X	X	0	Smart ArM
28		-	-	45	6:05	110	136	119	X	X	0	Smart ArM
29	U	11	13(k)	34	4:12	132	X	0	X	120	0	Touch Hand
30	V	12	13(l)	34	6:00	X	X	168	X	0	192	CyberTUM ARM
31		-	-	31	4:18	0	109	149	0	0	0	Viswajyothi
32		-	-	31	4:33	0	131	142	0	0	0	Viswajyothi
33		-	-	20	1:50	0	X	0	X	110	0	Touch Hands
34		-	-	20	1:54	0	X	0	X	114	0	Touch Hands
35		-	-	16	2:57	X	X	177	0	X	0	CyberTUM ARM
36	W	13	13(m)	15	2:31	0	151	0	0	0	0	Imperial ARM
37		-	-	14	1:01	61	0	0	0	0	0	Imperial ARM
38		-	-	14	1:18	78	0	0	0	0	0	Imperial ARM
39		-	-	0	0:01	X	X	X	X	X	0	CyberTUM ARM
					**Task:**	#1	#2	#3	#4	#5	#6	
					**Points:**	14	15	16	17	20	18	
					**Average time:**	65.73	105.87	96.50	87.86	73.81	71.89	
					**SD time:**	22.34	29.38	33.63	29.01	27.09	35.32	
					**Best time:**	42	57	51	63	42	40	
					**Abs diff best vs. average time:**	23.73	48.87	45.50	24.86	31.81	31.89	
					**Diff best vs. average time [%]:**	36.10	46.16	47.15	28.30	43.10	44.36	
					**Fails [%]:**	43.59	20.51	28.21	43.59	58.97	51.28	

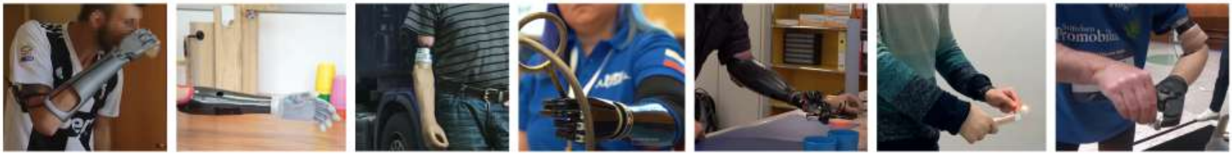

(a) (b) (c) (d) (e) (f) (g)

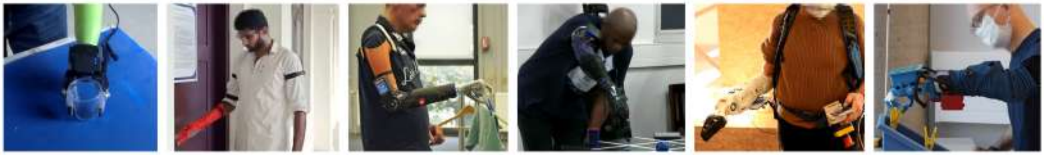

(h) (i) (j) (k) (l) (m)
