# Supplementary material for: Functional assessment of current upper limb prostheses: An integrated clinical and technological perspective
Source: PLoS One. 2023 Aug 16;18(8):e0289978. doi: 10.1371/journal.pone.0289978 (PMC10431634; doi:10.1371/journal.pone.0289978)
Supplement: S1 Appendix — (PDF) [file pone.0289978.s003.pdf]

Appendix S1 Cybathlon Task description, results and participants Tasks for Cybathlon 2016:

1. **Puzzle:** Pilots are asked to complete a puzzle-type task with several blue handles that differ in weight, size and shape to challenge different grips. The objects included are a plate, stick, ball, cord, hook, disk, cone, cylinder and a key.
2. **Wire loop:** Pilots hold a conductive wire loop with a blue handle with which a metal wire needs to be followed without touching the wire using the prosthetic arm only. This evaluate the wrist and more proximal joints capacity.
3. **Shelf and tray:** Various coomon objects (mostly blue elements) must be grasped from a shelf from different locations and drawers. These objects include an apple, banana, box with lightbulb, cereal keeper, one plate, one tea spoon, one knife, one fork, one tablespoon, one bowl and one coffee cup. This task evaluates the grasping in different body positions and the ability to complete maintenance work at home. Furthermore, a blue lightbulb needs to be screwed in to a table lamp.
4. **Breakfast table:** Breakfast must be prepared by cutting bread, unwrapping a sugar cube and opening a bottle, a jam jar and a can placed on a table. This task mostly evaluated the capability for dexterous bimanual interaction and precise grip force.
5. **Hang-up:** Clothes that are placed in a clothes hamper and need to be hung-up on a clothes rail by using hangers and blue clothespins. Moreover, two buttons of a blazer and a zipper of a jacket need to be closed. This task evaluates different finger motor skills, and wearability of the system.
6. **Carry bags:** Bags, parcels and balls must be carried over stairs, referring to daily life tasks. Placed on the floor, the objects released on a table.

Tasks for Cybathlon 2020:

1. **Breakfast:** A breakfast table must be prepared, equal to task #4 (cybathlon 2016). Furthermore, this time a candle must be lit.
2. **Laundry:** Hanging laundry is required, equal to task #5 (cybathlon 2016). In addition, shoes must be tied.
3. **Clean sweep:** A variety of blue objects must be grasped and moved individually from one table to a target position on the neighbouring table. This task tests the ability to cope with a diversity of requirements, such as different grip types, maintain grips during postural changes and the control of grip force.
4. **Home improvement:** Various tools and objects (mostly blue) must be used to complete crafting tasks. This task evaluates the prosthesis capacity to absorb vibrations and significant peak forces, as well as dexterous manipulation to use scissors or screwing a lightbulb.
5. **Haptic box:** 6 objects of specific shape and compliance must be identified in absence of any visual information, relying only on sensory feedback from the prosthesis (e.g. sounds, vibrations at the socket or haptic feedback). This task tries to test the availability of sensory feedback.
6. **Stacking:** In this task the pilots have to turn and stack blue cups into a vertical pyramid. This task specially evaluates the maintenance of a tight grip during postural changes of the arm (e.g. pronation and supination of the forearm, elbow flexion and extension).
